# Pertaining to Insurance

**Published:** 1876-10

**Authors:** 


The Secretary of the Chamber of Life Insurance reports the amount paid by twenty-seven companies during the month of August, 1876, upon policy claims, as follows:

| | |
|---|---|
| Losses by death | $1,520,828.83 |
| Matured endowments | 237,618.06 |
| Total | $1,758,446.89 |

We have received several communications asking for information relative to the "Lloyds Plate Glass Insurance Association" of this city, in reply to all of which we would say that so far as we have been able to learn this concern is *not* an insurance company, has no capital, is not subject to the restrictions of any law, and is not recognized by the Insurance Department of this State. On the other hand, it appears to be a sort of copartnership formed by individuals for the purpose of insuring plate glass, but on a plan which will exempt them from the operations of any law framed for the protection of the people against the ravages of fraudulent insurance companies. If the members of this "Lloyds" are responsible, as they claim to be, and if they chose to pay their losses when they occur, they answer all the purposes of a regularly organized insurance corporation; hence, while the insurance granted by the "Lloyds" may be reliable, we prefer the kind sold by regularly organized insurance companies, which are compelled to make annual statements of their financial condition.

---

Our correspondent, "Policy Holder," is informed that the Washington Life Insurance Co., of New York, is a perfectly sound and well-managed company.

---

Our correspondent, "C. B. S.," is informed that the company to which he refers has no license to transact business in the State of New York; hence, any policies issued here are illegal.

---

To "J. S. W." we would say the Mutual Protection Life Insurance Company of Philadelphia is classed as a co-operative concern without any substantial foundation and unworthy of confidence.

---

Stock of the Equitable Life Assurance Society, of New York, sold, a few days ago, at 453. What makes this stock so valuable? It certainly is not over dividend paying.

---

Two Binghampton (N. Y.) ladies, returning home from an evening entertainment, laid their hats upon a stove. Next morning they were found to have been burned up, and a claim was made for $1.50, under their insurance policy No. 1,512, on the item of wearing apparel. This was allowed and paid. The company honoring that loss is one of the largest in the country.

A newspaper paragraph says that the value of property insured in London is £540,000,000. Of the fires which occurred in London during the past five years four-fifths occurred to uninsured property.

